# Biopolymers from Natural Resources

**DOI:** 10.3390/polym13152532

**Published:** 2021-07-30

**Authors:** Rafael Balart, Daniel Garcia-Garcia, Vicent Fombuena, Luis Quiles-Carrillo, Marina P. Arrieta

**Affiliations:** 1 Technological Institute of Materials (ITM), Universitat Politècnica de València (UPV), Plaza Ferrándiz y Carbonell 1, 03801 Alcoy, Spain; 2 Departamento de Ingeniería Química y del Medio Ambiente, Escuela Técnica Superior de Ingenieros Industriales, Universidad Politécnica de Madrid (ETSII-UPM), Calle José Gutiérrez Abascal 2, 28006 Madrid, Spain; 3 Grupo de Investigación: Polímeros, Caracterización y Aplicaciones (POLCA), 28006 Madrid, Spain

During the last decades, the increasing ecology in the reduction of environmental impact caused by traditional plastics is contributing to the growth of more sustainable plastics with the aim to reduce the consumption of non-renewable resources for their production. Thus, in the last few years, the increased bio-based polymer production and application have positioned biopolymers as one of the most promising ways to meet the sustainable development goal of replacing traditional petroleum polymers with more sustainable materials in several industrial sectors.

In this context, the revalorization of agro-food wastes, biopolymers extracted directly from biomass such as polysaccharides, proteins and lipids, as well as those produced by yeast biomass, algae or by bacterial fermentation, have attracted a significant amount of interest, especially for medical devices, food packaging, agricultural films, membrane process applications, sustainable clothes and so on—interest which, despite successful recent developments in bio-based polymers up to the industrial scale, extends to their optimization for industrial exploitation. Therefore, significant attention has been paid to the improvement of those sustainable plastics directly derived from biomass overall performance to provide more than one advantageous property attributed to their versatility to be modified through chemical synthesis, copolymerization, and surface modification, as well as through the use of different additives (i.e., micro- and nanoparticles, plasticizers, and active agents, among others). Moreover, nowadays, the starting monomers or building blocks to obtain traditional plastics can be obtained from biomass instead of petrochemical resources, known as drop-in bioplastics. Drop-in bioplastics present the same advantages of traditional plastics in terms of performance and are more sustainable, while at the same time they can be directly transformed into the desired products by means of the same technology already available at the plastic industrial sector. Figure 1 summarizes several biobased polymers classified on the basis of their origin and obtainment process.

The present Special Issue gathers a series of twenty-four articles focused on the manufacturing and characterization of biopolymers and building blocks extracted from natural resources, as well as their potential scalability to the industrial level for several industrial applications. The Special Issue paid special focus on the improvement of the overall performance of biobased polymers by chemical modification process, the incorporation of sustainable additives such as biobased oligomers, by blending with another biobased or biodegrable polymeric matrix, as well as by the development of composites and nanocomposites through the incorporation of naturally occurring particles and nanoparticles.

Among other naturally derived biopolymers, polysaccharides are nontoxic and biodegradable, which increases their potential application in several fields. Polysaccharides, with cellulose as the most important representative since it is the most abundant polymer in nature and is found all around the world, are widely investigated. Although the most-used biopolysaccharides are obtained from plant origin (e.g., cellulose), they can be obtained from animal origin (e.g., chitin/chitosan) and also from microbial origin (e.g., bacterial cellulose). In this sense, the production of biobased polymers by using microorganisms has gained considerably attention during the last decade as a sustainable production method. In this regard, Irbe et al. [1] developed novel blends based on biopolysaccharides derived from biomass, softwood cellulose fibers such as softwood Kraft (KF) or hemp fibers (HF) and fungal fibers (hyphae). Regarding the fungal fibers, they concluded that among several fungal fibers, the fibers from screened basidiomycota fungi *Ganoderma applanatum* (Ga), *Fomes fomentarius* (Ff), *Agaricus bisporus* (Ab), and *Trametes versicolor* (Tv) were good for blending with softwood cellulose fibers. Thus, the fungal fibers were blended with KF and HF fiber pulp in different mass ratios (i.e., 50:50 and 33:33:33). The materials were characterized in terms of mechanical properties, air permeability and virus filtration efficiency. The overall performance of the hyphal polysaccharide/cellulose blends was highly affected by the microstructural features of each cellulosic material, while the ability of functional groups of hyphal polysaccharides to establish hydrogen bonding interactions with cellulose fibers was one of the key elements of network formation which determined the final blend properties and the potential applications. In this context, highly fibrillated hemp fibers showed the highest mechanical strength, while the blends containing Kraft fibers increased air permeability and showed a high virus reduction capacity. Thus, depending on the intended use the different microstructure of Hemp fibers or Kraft fibers will allow obtaining materials with interesting properties. For instance, materials with a low air permeability are interesting for food packaging applications where high gas barrier properties are of fundamental importance. Meanwhile, materials with a higher air permeability and virus filtration efficiency have potential for being used as gas permeable membranes, for example, as biobased filter layers in face masks. Despite its outstanding characteristics, cellulose in its native form is hard and provides some limitations for the plastic industry in terms of cellulose processing. Nevertheless, cellulose derivatives can overcome such problems. Thus, cellulose chemical modifications have been widely studied and nowadays several cellulose derivatives can be found commercially such as carboxymethyl cellulose (CMC), methyl cellulose (MC), hydroxyethyl cellulose (HEC), hydroxypropyl methylcellulose (HPMC), and cellulose acetate (CA).

Among others, CMC possesses good film-forming abilities because of the presence of a hydrophilic carboxyl group (–CH_2_COONa), which allows its dissolution in water. In this context, Rachtanapun et al. [2] revalorized coconut juice, a waste product from the coconut milk industry, into bacterial cellulose using *Acetobacter xylinum* to further obtain carboxymethyl cellulose (CMC) through chemical synthesis with different degrees of substitution. With this propose in mind, CMC was obtained from bacterial cellulose in the presence of monochloroacetic acid (MCA) via a carboxymethylation reaction and by simply varying the sodium hydroxide content (NaOH from 20% to 60%), the degree of substitution was controlled. The degree of substitution significantly increased with NaOH concentration up to 30% and then progressively decreased with further NaOH concentrations. As CMC is a water soluble cellulose derivative, the higher the degree of substitution, the higher the water vapor permeability. The flexibility of the material also increased with the degree of substitution. Thus, the developed method to modify cellulose from nata de coco allows tuning of the water permeability and the mechanical properties of the final material according to the need of the intended application by simple varying the NaOH content. CMC with different degrees of substitutions has been also obtained by Kluklin et al. [3] from the *Asparagus officinalis* stalk end, a typical herbal medicine used in Asia, by simply varying the NaOH content (from 20% to 60%) too, and it was also found that there is a higher degree of substitution for the NaOH 30% (*w*/*v*). CMC has been also used in the development of solid polymer electrolyte (SPE) film for sustainable energy storage systems due to its solubility in water and its naturally high degree of an amorphous phase that allows easier transport of conducting ions like lithium (Li^+^) and proton (H^+^). In this context, Sohaimy et al. [4] tuck this advantageous properties of CMC to develop CMC-based films for electrochemical applications. However, the inherent solid properties of the polymeric film impede ionic mobility leading to a low ionic conductivity, especially at room temperature. Thus, ammonium carbonate (AC), with two groups of ammonium ions, was used as a dopant in amounts from 1 wt.% to 11 wt.% with the main objective of injecting more H^+^ into the system and the CMC-AC thin electrolyte film was obtained by a solvent casting method. Both CMC and AC salts dissolved completely and had a low degree of crystallinity (X_c_) as it was demonstrated by X-ray diffraction (XRD). Although, the ionic conductivity for a biopolymer electrolyte intended for commercial energy storage technology needs to be at least a minimum value of ~10^−4^ Scm^−1^, they obtained promising results since the highest ionic conductivity obtained for CMC doped with 7 wt.% was 7.71 × 10^−6^ Scm^−1^, resulting in higher results than other diammonium salts tested (i.e., ammonium adipate and ammonium sulfate), showing that further enhancement is still needed to increase the ionic conductivity for suitable electrochemical applications (~10^−4^ Scm^−1^) and the CMC-AC films show promising prospects for electrochemical applications.

Panraksa et al. [5] developed orodispersible films (ODF) based on hydroxypropyl methylcellulose (HPMC: E5 and E15) by extrusion-3D printer technology to enhance the solubility and dissolution of poorly water-soluble drugs (i.e., phenytoin). ODFs are required to rapidly disintegrate in the buccal cavity (i.e., within a minute), without requiring water, thus HPMC, which is a well-known hydrophilic and film-forming polymer, is an ideal candidate for this propose. HPMC E5 and E15 were used as the film-forming polymers, while propylene glycol and glycerin were used as plasticizers. They optimized the processing conditions and the results showed that the phenytoin-loaded HPMC E15 (at a concentration of 10% *w/v* of HPMC E15) was the most suitable formulation of HPMC, since it exhibited a good physical appearance, good mechanical strength, low Young’s modulus and high elongation at break, rapid in vitro disintegration time (within 5 s) as well as a rapid drug release (up to 80% within 10 min), showing the improvement of solubility and dissolution rate of phenytoin at the same time as allowing ease of handling and application.

Another widely used cellulose derivative is cellulose acetate (CA). For instance, it has been used by Arrieta et al. [6] as a carrier of *Cucumis metuliferus* fruit extract to provide to corona-treated low-density polyethylene (LDPE) films with antioxidant activity by coating a CA-based polymeric solution, containing different amounts of *Cucumis metuliferus* fruit extract (1, 3 and 5 wt.%), onto the LDPE film. The materials showed antioxidant activity (between 0.3 and 0.6 mg Trolox/dm^2^) after performing migration studies in a fatty food simulant and also showed their ability to reduce the browning effect of fresh-cut apples in direct contact. Additionally, the coating not only provided an antioxidant active functionality, but also improved the oxygen barrier performance to the final bilayer material, showing their use in active food packaging applications.

Another interesting approach to obtain a solution of insoluble cellulose is regenerated cellulose (RC). RC refers to the chemical dissolution of insoluble natural cellulose followed by the recovery of the material from the solution by means of different methods, such as cellulose carbamate processes, lyocell processes, and viscose processes. In this context, Husseinsyah et al. [7] used an ionic liquid (1-butyl-3-methylimidazolium chloride) as a novel method to obtain RC from empty fruit bunches (EFB) to develop a self-reinforced composite for biodegradable packaging applications. They studied the possibility of improving the interphase bonding between the cellulosic matrix and the filler in the self-reinforced composite by means of EFB chemical treatment with a methyl methacrylate (MMA) chemical treatment. They observed that the substitution of the –OH group of the EFB cellulose with the ester group of the MMA allowed the dissolution process of the EFB during the regeneration process owing to weakening of the hydrogen bonding of the cellulose. This allowed us to obtain a more crystalline and homogeneous EFB RC biocomposite film, with an improved thermal stability and mechanical performance. Another very important cellulosic material is cotton, which is widely distributed worldwide. It has a higher yield compared to other commercial crops, and thus several products are obtained from cotton seed, such as oil. In this context, Chen et al. [8] used bio-based cottonseed oil (CO) and two of its derivatives, fatty acid of cottonseed oil (COF) and sodium soap of cottonseed oil (COS), to tune the density and mechanical strength of polysulfide polymers by means of a free radical addition mechanism. COF reacts with sulfur to generate serials of polysulfide-derived polymers with a lesser viscosity and tensile strength. Whereas COS was not involved in the reaction with sulfur, as a consequence of the high melting point of sodium linoleate and sodium oleate. Nevertheless, COS it was able to increase the density and tensile strength of polysulfide-derived polymers when it was used as a filler. Moreover, the developed polysulfide-based polymers showed good reprocessability and recyclability, showing their potential as bio-based functional supplementary additives.

Ammar et al. [9] developed a novel adsorbent material for cationic dyes based on a polyelectrolyte multi-layered (PEM) system cross-linked to a cellulosic-based material. The PEM was formed by an alternation of layers of two polysaccharides, sodium alginate polyanion and reticulated citric acid with k-carrageenan, thus providing many carboxylate and sulfonate groups grafted on the cellulosic surface. The developed system provides outstanding adsorption capacities for cationic dyes (e.g., with a capacity above 522.4 mg/g for methylene blue).

The processing and revalorization of lignocellulosic materials obtained from agro-food wastes, as well as the extraction through sustainable approaches, have gained considerable interest. Gómez-Patiño et al. [10] extracted cellulosic cutins from of *S. aculeatissimum* and *S. myriacanthum* fruit peels, using enzymatic (i.e., *A. niger pectinase*, *A. niger cellulose* and *A. niger hemicellulose*) and chemical (trifluoroacetic acid hydrolysis) methods. They selected these fruits since they are not for human or animal consumption and are thus interesting as a potential raw material to produce sustainable materials. The hydrolyzed cutins showed mainly 10,16-dihydroxyhexadecanoic acid (10,16-DHPA) monomers composition and the obtained films showed a good homogeneity.

Lignocellulosic derivatives have also gain considerable interest as micro and nanofillers. In this context, Adamcyk et al. [11] studied the extraction of lignin from wheat straw, spruce, and beech using ethanol organosolv pretreatment at temperatures from 160–220 °C, and further precipitated by solvent-shifting obtaining lignin micro- and nanoparticles (with mean hydrodynamic diameters from 67.8 nm to 1156.4 nm). They observed that higher pretreatment temperatures increased the delignification of the raw materials but also favor depolymerization and structural alteration of the extracted lignin, increasing the particle sizes as well as agglomeration at pretreatment temperatures of over 200 °C. The obtained particles were then purified by dialysis and the showed interesting antioxidant activity (i.e., from 19.1 and 50.4 mg Lignin/mg ascorbic acid equivalents).

On the other side, lignocellulosic nanoparticles have been obtained from yerba mate (*Ilex paraguariensis*) waste, a typical infusion widely consumed in Latin America (i.e., Argentina, Brazil, Uruguay and Paraguay), by means of an aqueous extraction procedure (with mean average size of 495 nm). The obtained nanoparticles were used by Beltrán et al. [12] to reinforce mechanically recycled bioplastic (i.e., polylactic acid). Poly(lactic acid) (PLA) is chemically synthesized starting from simple sugars obtained from biomass fermented to lactic acid. It is one of the most promising biopolyesters for massive industrial applications due to its availability in the market at a competitive cost, and it can be recycled following the mechanical recycling process used for other traditional plastics (e.g., polyethylene terephthalate, PET). Low amounts of yerba mate nanoparticles (i.e., 1 wt.%) were able to increase the intrinsic viscosity and to improve the oxygen barrier properties of mechanically recycled PLA, showing the interest of such nanocomposites in the food packaging field. Ramos et al. [13] also studied PLA-based nanocomposites for food packaging applications. In this case, PLA was loaded with commercial montmorillonite (D43B) at two different concentrations (2.5 and 5 wt.%) and was further incorporated with thymol as an antioxidant and antimicrobial agent for active food packaging applications. The results showed that the addition of 2.5 wt.% D43B and 8 wt.% thymol leads to a material with a good balance of properties, with antibacterial activity against *Escherichia coli* and *Staphylococcus aureus.*

Another interesting source for sustainable additives are seeds fruits, peel and/or nut’s shells, from which can be obtained flours and natural particles and/or nanoparticles, as well as vegetable oils. In this regard, Jorda-Reolid et al. [14] obtained micronized Argan shell (MAS) particles from residues of the *Argania spinosa* plant, a tropical plant whose fruit is used in Morocco to prepare oil. The authors used MAS particles (with mean average long of 70 µm and mean average wide of 45 µm) as reinforcing fillers of high-density polyethylene obtained from sugarcane (Bio-HDPE). Meanwhile, to improve the low compatibility between MAS and Bio-HDPE, they studied two compatibilizing agents, polyethylene-grafted maleic anhydride (PE-g-MA) and maleinized linseed oil (MLO); as well as halloysite nanotubes (HNTs), a second reinforcing filler. The obtained materials showed improved stiffness, high ductility, and good thermal stability, as well as a visual appearance very similar to that of reddish-color woods, showing their interest as wood plastic composites with the additional advantage of being chipper rather than neat Bio-HDPE. González Martínez et al. [15] optimized the carbonation reaction of epoxidized linseed oil (ELO) with carbon dioxide (CO_2_) in the presence of tetrabutylammonium bromide (TBAB) as catalysts, to obtain carbonated vegetable oils (CVOs), which have potential applications as interesting starting materials for the formation of polymers (e.g., monomers, additives, lubricants and plasticizers), particularly in the synthesis of non-isocyanate polyurethanes (NIPUs). They were able to obtain large conversion (96%), carbonation (95%), and selectivity (99%) at low temperature (90 °C), and thus the high carbonate content obtained for carbonated ELO showed their promising performance for the synthesis of NIPU with the required properties and more sustainable characteristics. On the other hand, Dominici et al. [16] studied the possibility of obtaining bioplastics from wheat flour particles as a novel source and an energetically and economically cheap alternative to other thermoplastics. The refined flours, with different contents of bran, were first plasticized with glycerol and then the authors attempted to find how different contents of grinded bran could affect the deformability of the flour by blending with low melting polymeric fractions (such as poly(ε-caprolactone) (PCL), and polybutylene-adipate-*co*-terephthalate (PBAT)). They studied different processing parameters and they were able to obtain a novel thermoplastic wheat flour (TPWF), which showed an improved performance when glycerol was partially replaced by water and when it was blended with PCL, as well as adding citric acid as a compatibilizer.

Ferrándiz et al. [17] studied the potential use of soy protein (SP) fibers functionalized with an undisclosed antimicrobial agent as well chitin (with inherent antimicrobial properties) fibers as sustainable active textiles to replace synthetic polymers widely used in this sector. The thermal resistance of both weft-knitted fabrics was similar to that of cotton, whereas their air permeability was higher, particularly in the case of chitin due to its higher fineness, which makes these natural fibers very promising for summer clothes.

After cellulose, starch is the second most abundant polymer in nature and, thus, has been widely studied for several sustainable industrial applications. Zhang et al. [18] developed potato starch-based foams by means of a microwave treatment. They blended potato starch with chitosan as a reinforcing phase and it was found that starch-based foams with a larger proportion of starch showed a small pore size and a low density with higher compressive strength ascribed to the good compatibility between both polymeric matrices due to the formation of hydrogen bond interactions between amino groups of chitosan and hydroxyl groups of starch. Those interactions were also responsible to maintained intact the morphological structure of the foam in water for 10 days, while the starch-based foams completely degraded in water after 30 days. Thus, the developed starch/chitosan foams resulted in interesting results for their use as active materials in biomedical applications as well as in drug and food packaging.

For several industrial applications, starch is particularly used in its thermoplastic form, known as thermoplastic starch (TPS). To obtain TPS, native starch requires disruption of the granule organization by means of a combination of high water content and heat, leading to the starch granule swelling and consequently starch gelatinization. In this regard, Crucean et al. [19] studied the gelatinization process of three native starches: (i) wheat, (ii) potato, and (iii) waxy corn starch, as well as starch from a wheat flour in mixtures of water and choline chloride (an ionic compound, which has a “structure making” effect). They demonstrated that choline chloride/water system exhibits an allotropic change at low water concentrations and solubilization for water contents greater than 30%. They observed a stabilization, or a better organization, of the ordered regions of the starch in the presence of choline chloride as it was demonstrated by an X-ray diffraction analysis in a heating cell that the crystalline rearrangement of the structure of the starch grain takes place simultaneously with the solubilization phenomenon of amylose.

Osman et al. [20] studied thermoplastic starch (TPS)-based biocomposites using dolomite (DOL) as a filler in its pristine form (DOL(P)), as well as dolomites, after a simple and scalable sonication process (DOL(U)). TPS-DOL biocomposites intended for packaging applications were prepared at different loadings (i.e., 1, 2, 3, 4 and 5 wt.%). The TPS-based biocomposites with a high dolomite loading (i.e., 4 and 5 wt.%) showed better mechanical performance, showing greater tensile and tear properties, particularly in the case of biocomposites loaded with a sonicated process assisted by dolomite due to a reduction in particle size allowing their better dispersion within the TPS matrix. Thus, the high abundancy and low cost of dolomite, in combination with the simple, scalable and environmental friendly method of sonication, showed their interest to be of use as reinforcing fillers for TPS to produce a sustainable biocomposite for packaging applications. Although biomass-derived biopolymers are mainly extracted from plants, they can also be obtained from animal resources. For instance, gelatin has been traditionally used in the development of soft capsules (softgels) due to its biodegradable nature, as well as its ability to form thermo-responsive hydrogels, inspiring interest in the biomedical sector and food industry. In recent years, new materials have been explored in the food industry to partially or completely replace gelatin with other non-animal natural hydrocolloids. In this context, Otálora et al. [21] developed gelatin/cactus mucilage softgel capsules to encapsulate oil extracted from sacha inchi (*Plukenetia volubilis L.*) seeds, a rich source of polyunsaturated fatty acids (PUFAs) that are beneficial to human health. They submitted the softgel capsules to an in vitro digestion process to simulate gastric conditions and the protective capacity of the gelatin/cactus mucilage-based softgel against digestive processes was evaluated. Although the study revealed a reduction in the content of polyunsaturated fatty acids (PUFAs) after the digestion process and, thus, a reduction of the nutritional value, they concluded that gelatin/cactus mucilage microcapsules can act as interesting bioactive delivery systems for acidic food (e.g.,: fruit juices or dairy drinks) before being subjected to digestive processes.

As has already been mentioned, the biobased polymer obtainment, by means of microorganisms, has gained a lot of interest. In this context, the family of polyhydroxyalkanoates (PHAs) are polyesters biologically synthesized by controlled bacterial fermentation in response to nutrient limitation as an intracellular storage of food and energy. The homopolymer, poly(hydroxybutyrate) (PHB), is the simplest and most common representative of the PHA family, but PHAs also comprise many copolyesters, polyhydroxybutyrate-*co*-hydroxyalkanoates, that have gained high industrial interest in the bioplastic sector, such as polyhydroxybutyrate-*co*-hydroxyvalerates (PHBV), or polyhydroxybutyrate-*co*-hydroxyhexanoate (PHBH), polyhydroxybutyrate-*co*-hydroxyoctonoate (PHBO), and polyhydroxybutyrate-*co*-hydroxyoctadecanoate (PHBOd). For instance, Ivorra-Martinez et al. [22] developed PHBH/PCL blends with different compositions ranging from 0 to 40 of PCL wt.% by means of extrusion process followed by injection molding, with the main objective of reducing the inherent embrittlement of PHBH produced due to the aging process (i.e., secondary crystallization). Although they mainly found a lack of miscibility between both polymeric matrices, as revealed by the thermogravimetric analysis, they concluded that the addition of high amounts of PCL (i.e., 40 wt.%) contributed to an increase in ductility and to a decrease in the typical brittleness of PHA. PCL considerably improved the toughness, as well as the impact resistance, of neat PHBH. Therefore, PHBH/PCL blends showed their suitability for the packaging industry. Then, Ivorra-Martinez et al. [23] developed Wood Plastic Composites (WPCs) based on PHBH loaded with lignocellulosic particles of almond shell flour (ASF), a by-product from the agro-food industry, by means of extrusion process followed by injection molding. Since the addition of ASF (with a maximum particle size of 150 µm) leads to an embrittlement and reduced toughness of the WPC, they used oligomeric lactic acid (OLA) to provide improved properties to the final PHBH-ASF/OLA material. Interestingly, a remarkable increase in impact strength with 20 phr OLA addition was achieved. In fact, OLA provides PHBH polymer chains of a high mobility due to a plasticizing effect, leading to an improvement in toughness, even on composites with 30 wt.% ASF. Additionally, OLA decreased the water absorption capacity of WPC, thus broadening potential industrial applications of PHBH-ASF/OLA in high humidity environments.

As it was previously mentioned, nowadays many traditional plastics, which were obtained for decades through the classic petrochemical production routes, have recently found “green” routes for their production (e.g., Bio-PE, Bio-PET, etc.). In this regard, polyamide 6 (PA6) can be replaced with a biobased variant that exhibits a high renewable content and with very similar properties known as PA610. In this context, Marset et al. [24] used PA610 partially biobased polyamide (Bio-PA) to develop nanocomposites reinforced with 10%, 20%, and 30% halloysite nanotubes (HNTs) as a natural flame retardancy filler by means of an extrusion process, followed by injection molding. The results showed that HNTs promote a significant reduction in the optical density and in the number of toxic gases (i.e., CO_2_) emitted during combustion, especially when PA610 was loaded with 30% HNTs. Thus, the nanocomposites showed good flame retardancy properties.

In summary, the Special Issue Biopolymers from Natural resources published in *Polymers*, compiles the recent research works in biopolymers obtained from natural resources and the strategies to improve their overall performance for sustainable industrial applications. From the above, improvements of biobased polymers was successfully achieved by means of copolymerization, blending, the use of compatibilizers, or plasticizers (vegetable oils, oligomers, etc.), as well as with naturally occurring or naturally derived particles on the micro- and nano-scale. Special interest has been shown to the revalorization of agro-food wastes for the extraction/production of additives or polymers with an interest in the industrial sector. The resulting, more sustainable materials can find potential applications in several industrial sectors, such as in the controlled release of active compounds for active food packaging, bioactive delivery systems, or biomedical devices (e.g., soft capsules, etc.), textiles (e.g., sustainable clothes) or as wood plastic composites (e.g., furniture and outdoor applications).

Future research efforts on biobased polymer extraction and production methods, as well as the optimization of their final production into co-polymers, blends, composites, and nanocomposites, are still needed to properly transfer the proposed developments, as well as future ones from the laboratory scale level to the industrial sector to reach more environmentally friendly materials within a circular economy approach.

## Figures and Tables

**Figure 1:**
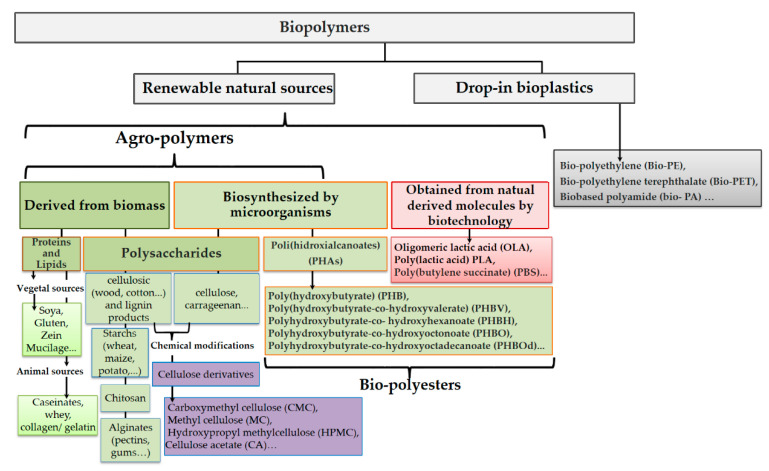
Classification of biobased polymers.
